# Preventing Antimicrobial Resistance Together: Reflections on AMR Week 2023

**DOI:** 10.1007/s44197-023-00178-1

**Published:** 2024-01-08

**Authors:** Jaffar A. Al-Tawfiq, Shahul H. Ebrahim, Ziad A. Memish

**Affiliations:** 1 Infectious Disease Unit, Specialty Internal Medicine, Johns Hopkins Aramco Healthcare, Dhahran, Saudi Arabia; 2 Division of Infectious Diseases, Indiana University School of Medicine, Indianapolis, IN USA; 3 Division of Infectious Diseases, Johns Hopkins University, Baltimore, MD USA; 4 University of Sciences, Technique and Technology of Bamako, Bamako, Mali; 5 King Saud Medical City, Ministry of Health & College of Medicine, Alfaisal University, Riyadh, Saudi Arabia; 6 Hubert Department of Global Health, Rollins School of Public Health, Emory University, Atlanta, GA USA; 7 Kyung Hee University, Seoul, South Korea

**Keywords:** AMR, Antimicrobial resistance

Human life expectancy improved worldwide during the last century largely attributable to foundational changes in health enabling factors such as clean drinking water, improved sanitation, and the discovery of antibiotics. Of these three developments, antibiotics are both a blessing and a challenge to continued human prosperity. The overuse and misuse of antibiotics, antivirals, and antifungals have led to the emergence of resistant strains of bacteria, viruses, and fungi. Antimicrobial resistance (AMR) is not a distant, abstract menace; it is a clear and present danger in slow motion, with implications to both human and animal health.

The World AMR Awareness Week 18-24 November is a global campaign to raise awareness and understanding of AMR and promote best practices among One Health stakeholders to reduce the emergence and spread of drug-resistant infections. It is encouraging to see that the theme from the previous year—"Preventing antimicrobial resistance together"—remains relevant for the AMR Week 2023. This theme underscores the need of our joint efforts to protect the efficacy of antimicrobial medications and the shared responsibility we have in the fight against AMR.

The implication of AMR extends beyond clinical medicine. Antimicrobial resistance affects healthcare systems, economies, and patient outcomes globally, with far-reaching effects. The World Health Organization (WHO) estimates that AMR causes 700,000 deaths annually; if unchecked, this number could increase to 10 million deaths annually by 2050 [1]. Beyond just harming people, AMR also has an impact on the environment, food security, and animal health. Treatment for infections brought on by resistant microbes is more challenging, which raises the risk of prolonged illness, higher healthcare expenses, and higher death rates. AMR has a significant financial cost; estimates indicate that, in the absence of intervention, the global economy could lose $1 trillion in total by 2050 [2, 3]. It is also estimated that AMR is associated with 4.95 million (3.62–6.57) deaths in 2019 overall, including 1.27 million (95% UI 0·911–1·71) deaths attributable to bacterial AMR [4].

There are multiple reasons to fight AMR. Antimicrobial medications are crucial for treating a variety of infectious diseases, such as bacterial, fungal, and some viral infections. Nevertheless, the efficacy of these therapies is in jeopardy due to the rise and dissemination of antibiotic resistance. By addressing AMR, we can maintain the effectiveness of currently available antimicrobial medications, guaranteeing their continued ability to fight infections and save lives. Treatment failures, protracted illnesses, and higher death rates are all consequences of AMR. When infections develop resistance to first-line antibiotics, medical professionals are forced to use more potent, costly, and possibly hazardous medications. There may occasionally be no effective treatments available, which raises morbidity and death rates. Addressing AMR will help us avert a future where common infections become life-threatening once again.

AMR puts a heavy strain on global healthcare systems. Healthcare resources are under pressure and costs are rising due to the need for more expensive treatments, longer hospital stays, and more thorough diagnostic testing. We can lessen the strain on healthcare systems and make sure that resources are distributed sustainably and effectively by battling AMR. In veterinary medicine, antimicrobial medications are also essential for treating animal infections. AMR develops and spreads as a result of the overuse and misuse of antibiotics in livestock production. Food security, animal health, and the reduction of animal-to-human AMR transmission can all be achieved by encouraging the responsible use of antibiotics in agriculture and animal husbandry.

AMR has a substantial negative macroeconomic and microeconomic impact on nations. The financial burden incurred by AMR-related decreased economic output, lost productivity, and higher healthcare costs is significant. We can safeguard economic stability, promote sustainable development, and lessen the negative financial effects connected to this threat to global health by tackling AMR.

Developing successful strategies requires a thorough grasp of AMR patterns and trends. The emergence and dissemination of resistant microbes can be tracked through the collection and exchange of data made easier by programs such as the Global Antimicrobial Resistance Surveillance System (GLASS) [5]. Cooperation among nations and organizations can improve data quality, standardize surveillance techniques, and encourage timely information sharing. Maintaining the efficacy of antimicrobial medications requires their responsible use. Implemented in a variety of healthcare settings, antimicrobial stewardship programs aim to maximize the use of antibiotics while enhancing diagnostic capabilities and encouraging infection prevention and control strategies. We can minimize the emergence of resistance and cut down on the number of needless prescriptions written by teaching the public and medical professionals about the proper use of antibiotics [6].

Even with tremendous efforts made to increase public awareness of AMR, larger audiences still need to be reached. Education campaigns aimed at the public, legislators, and healthcare professionals are crucial in bringing attention to the effects of AMR (6). Preventing the spread of resistant infections requires the implementation of strong infection prevention and control practices. However, these efforts are hampered by a lack of resources, poor infrastructure, and inconsistent adherence to guidelines, especially in settings with limited resources [7]. To address these issues, it will be necessary to make investments in healthcare infrastructure, train healthcare professionals, and guarantee that patients have access to necessary resources.

Of note, regulation and restriction of antibiotic prescription and sales are a huge challenge in resource poor countries. Given that AMR in one country ultimately extends to other countries, this situation negates the gains achieved in developed nations through stringent antimicrobial prescription and sales controls. Given the implication of individual responsibility of clinicians and pharmacists, AMR prevention should be integrated to relevant academic modules and continuing education programs. Effective coordination is difficult to achieve, though, because of differences in national policies, the complexity of global governance structures, and inequalities in healthcare systems. [8]. To effectively combat AMR, cross-sector and national coordination of efforts is crucial.

The idea of "One Health" is essential in the fight against AMR [9]. A thorough grasp of the elements influencing the spread of AMR is made possible by integrated surveillance systems that track AMR in humans, animals, and the environment. These systems also enable the early detection of emerging resistance trends. We can create focused interventions and preventive measures by collaborating and exchanging data between veterinary and human medicine (CDC, 2020). One Health acknowledges the function of animals as reservoirs of resistant genes and bacteria, including pets, livestock, and wildlife [10, 11]. One of the main components of One Health approaches is encouraging antimicrobial stewardship practices in both human and animal healthcare settings. This entails using antibiotics responsibly, maximizing dosage schedules, enhancing diagnostic capabilities, and putting infection prevention and control strategies into action. It is possible to minimize the selection pressure for resistance, minimize the needless use of antimicrobials, and maintain the efficacy of currently available medications by implementing uniform guidelines and best practices in both human and veterinary healthcare.

Professionals from various disciplines are encouraged to collaborate and communicate with one another through One Health. Stakeholders can develop comprehensive strategies to combat AMR and exchange knowledge and best practices by fostering interdisciplinary partnerships [12]. Research institutions, governmental and non-governmental organizations, and international bodies can all collaborate to facilitate the sharing of knowledge, resources, and data. This includes providing funding for studies aimed at comprehending the mechanisms behind AMR, creating novel antimicrobials, diagnostic tools, and alternative treatments, as well as investigating cutting-edge methods of infection prevention and control. Included also are guidelines for surveillance and reporting, rules on the use of antimicrobials in agriculture and aquaculture, and regulatory frameworks to support responsible use of antibiotics in human and veterinary medicine.

Stakeholders worldwide will have the chance to reaffirm their shared commitment to preventing antimicrobial resistance during AMR Week in 2023. We can fortify our resolve to act as a group by acknowledging the benefits of combating AMR, which include the maintenance of efficacious treatments, fostering antimicrobial stewardship, the avoidance of elevated mortality and morbidity, the defense of healthcare systems, the preservation of food security, and the maintenance of economic stability. To ensure a healthier and more resilient future for all, it is imperative that challenges be overcome and collaboration be fostered at local, national, and international levels.

## Abbreviations

AMR: Antimicrobial resistance
WHO: World Health Organization
GLASS: Global Antimicrobial Resistance Surveillance System
CDC: Centers for Disease Control and Prevention in the USA
